# Ensuring Comprehensive Care: An Audit on Physical Parameter Monitoring in Children and Adolescents With ADHD on Medication

**DOI:** 10.1192/bjo.2025.10671

**Published:** 2025-06-20

**Authors:** Rakesh Sharma

**Affiliations:** CAMHS St Ann’s Hospital, London, United Kingdom

## Abstract

**Aims:** In child and adolescent psychiatry, particularly within Child and Adolescent Mental Health Services (CAMHS), monitoring the physical health of patients on ADHD medications is of paramount importance. Medications such as stimulants can have significant impacts on physiological processes, including cardiovascular health. This audit aims to assess current practices in monitoring physical parameters in children and adolescents prescribed ADHD medication at St Ann’s Hospital (Haringey), identify gaps in practice, and recommend improvements based on recent research.

**Methods:** Study design: Retrospective audit.

Sample: Medical records of children and adolescents diagnosed with ADHD and currently on medication.

Data Collection: Frequency and documentation of weight, height, blood pressure, and heart rate measurements over the past year.

Analysis: Descriptive statistics will be used to compare current practices against established standards.

Ethical considerations: Ensured patient confidentiality throughout the audit.

Literature review.

**Results:** Of the 50 clients reviewed:

46 clients had their blood pressure, pulse rate, height, and weight measured at every appointment.

2 clients were referred for shared care, impacting tracking of their monitoring.

1 client’s height was not checked at appointments, though other parameters were monitored.

1 client did not have blood pressure and pulse rate monitored during follow-up.

**Conclusion:** The audit highlighted significant adherence to monitoring standards but identified gaps in certain areas. Implementing the recommendations and maintaining a strong commitment to regular audits will enhance the quality of care provided to children and adolescents with ADHD in CAMHS. A re-audit will be planned to evaluate the impact of changes made from this audit.

